# Eddy Effect and Dynamic Response of High-Speed Solenoid Valve with Composite Iron Core

**DOI:** 10.3390/ma16175823

**Published:** 2023-08-25

**Authors:** Peng Liu, Ruqin Zhang, Qing Zhao, Shijian Peng

**Affiliations:** College of Automotive and Mechanical Engineering, Changsha University of Science & Technology, Changsha 410114, China

**Keywords:** high-speed solenoid valve, composite iron core, Eddy effect, dynamic response

## Abstract

To alleviate the Eddy effect of the high-speed solenoid valve (HSV) and improve its dynamic response speed, a novel HSV with a composite iron core is presented. The time-step finite element method is used to establish and verify the numerical simulation of HSV coupling multiple physical fields. Then, the Eddy effect and dynamic response characteristics of the conventional and composite HSVs are further compared and analyzed. The results showed that the Eddy current loss in the main pole was the largest for the conventional HSV, accounting for 72.5% and 64.4% in the actuation and release processes, respectively. It was found that the Eddy effect of the composite HSV was obviously weakened, and the total Eddy current losses in the actuation and release processes were reduced by 58.8% and 38.7%, respectively. Meanwhile, the actuation response time and release response time of the composite HSV were shortened by 15.6% and 18.5%, respectively. In addition, increasing the peak voltage further shortened the actuation response time of the composite HSV, but had no significant effect on the response time of the conventional HSV.

## 1. Introduction

As an automatic control component, the high-speed solenoid valve (HSV) has been widely used in hydraulic and pneumatic fields because of its simple structure, fast response speed, low price, and high reliability [1,2,3,4,5]. Such applications include the fuel quantity control of the electronic fuel injection system [6,7,8,9,10,11], the pressure adjustment of the automobile brake system [12,13,14], the process control of material processing [15,16], etc. Faster dynamic response speeds of HSVs can realize the more accurate adjustment of flow and pressure of the controlled system [17,18]. Therefore, it is of great significance to improve the dynamic response characteristics of the HSV. Previously, several academics have achieved significant advancements in the HSV field.

Fan et al. investigated the effect of the punch position and size on the dynamic response of the HSV and implemented a multi-objective optimization of the fan groove geometric parameters and the armature thickness; then, the actuation and release response times were reduced by 11.1% and 30.0%, respectively [17]. Hung et al. found that optimizing the coil’s cross-sectional shape and relative position to the plunger can obtain a large electromagnetic force and short response time for the HSV [19]. Yang et al. analyzed the effects of key structural factors on the static electromagnetic characteristics of the HSV and optimized five key influence parameters by the Taguchi method [20]. Li et al. carried out the multi-objective optimization for the actuation and release response times of the HSV based on response surface methodology and NSGA-II (non-dominated sorting genetic algorithm II), and after optimization, the actuation and release response times were reduced by 17.7% and 37.4%, respectively [21]. Zhao et al. investigated the influence of the driving current and essential structural parameters on the static electromagnetic force of the HSV and concluded that the changes in the electromagnetic force were determined by the total magnetic reluctance and the range of the driving current [22]. Wang et al. revealed the influence rules of four parameters (i.e., the firing current, holding current, spring pre-tightening force, and spring stiffness) on the dynamic response characteristics of the HSV [23]. Liu et al. proposed a novel HSV with a permanent magnet based on the principle of the parallel magnetic circuit, and they obtained the optimal design solution by response surface methodology and a genetic algorithm, significantly reducing the power consumption and coil loss and improving the dynamic response speed of the HSV [24].

In the above research, the influence and the effect of the optimization of structural and driving parameters on the dynamic response characteristics of the HSV were studied in depth. Since the iron core and armature of the HSV are composed of soft magnetic materials with high magnetic permeability and certain conductive properties, the Eddy effect will occur in the excitation process of the HSV. This phenomenon inhibits the establishment and decay of the magnetic field and slows down the growth and decay of the electromagnetic force, reducing the dynamic response speed of the HSV [25]. Additionally, it generates heat, reducing the reliability of the system. Therefore, many scholars have paid much attention to the Eddy effect and its influence on the dynamic response characteristics of the HSV. Zhao et al. [26], Bai et al. [27], and Tan et al. [28] conducted numerical simulations to find that the Eddy effect has an important impact on the dynamic response of the HSV. Moreover, Zhao et al. found that the Eddy effect exerts a greater influence on the actuation response time than the release response time for the conventional HSV. Bai et al. reported that the Eddy effect has a larger impact on the release response time than on the actuation response time for the HSV with a permanent magnet. Meanwhile, Zhao et al. analyzed the influence of drive voltage and the maintenance current on the Eddy effect and dynamic response, and they concluded that a high drive voltage and maintenance current could improve the dynamic response speed of the HSV but would lead to increased Eddy current loss and reduced energy conversion efficiency [29,30]. Cheng et al. [31,32] and Zhong et al. [33] established that the Eddy current loss of the HSV can be reduced and its dynamic response speed can be improved by optimizing the drive strategy. Cheng et al. [34] designed an HSV with an Fe-based nano-crystalline soft magnetic alloy using metal injection molding technology, which improved the performance of soft magnetic materials, reduced the Eddy current loss of the HSV, and improved the dynamic response speed. Dai et al. [35] and Zhao et al. [25,26] found that grooving on the iron core can suppress the Eddy effect to a certain extent and enhance the dynamic response speed of the HSV.

In fact, the use of high-resistivity soft magnetic materials has proved the most direct and simplest way to reduce the Eddy effect. However, not only the growth rate of the electromagnetic force but also its size would affect the dynamic response speed of the HSV. The upper limit of the electromagnetic force depends on the saturation flux density of soft magnetic materials. Thus, the ideal soft magnetic material for the HSV should have high resistivity and high saturation flux density, but it is difficult to achieve both [36]. Therefore, in order to further improve the dynamic response speed of HSVs, this paper proposes a novel HSV with a composite iron core (hereinafter referred to as “composite HSV”), which combines the characteristics of different soft magnetic materials. Then, the multi-physics coupling numerical simulation models are established. Finally, the Eddy effect and dynamic response characteristics of conventional and composite HSVs are compared and analyzed, and the distribution raw and characteristics of the Eddy current loss of the HSV are revealed. The findings provide a theoretical reference for the design of an HSV with a high response and low power consumption and help to improve the control accuracy of a system controlled by the HSV.

## 2. Structure and Principle

**Figure 1:**
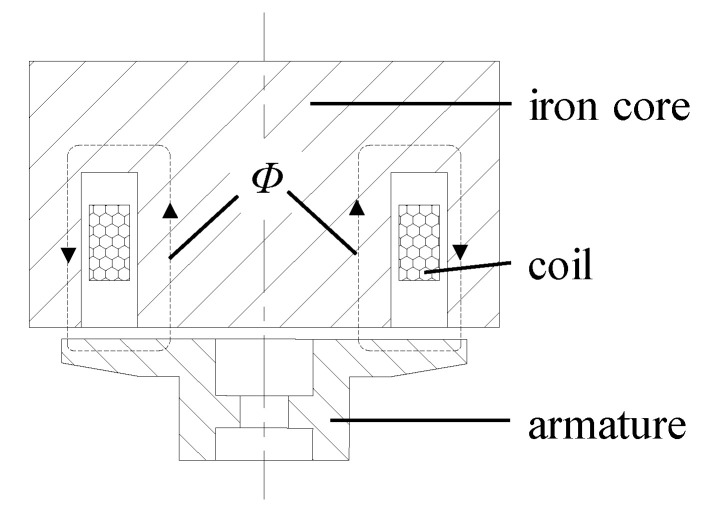
Structure diagram of conventional high-speed solenoid valve (HSV).

**Figure 2:**
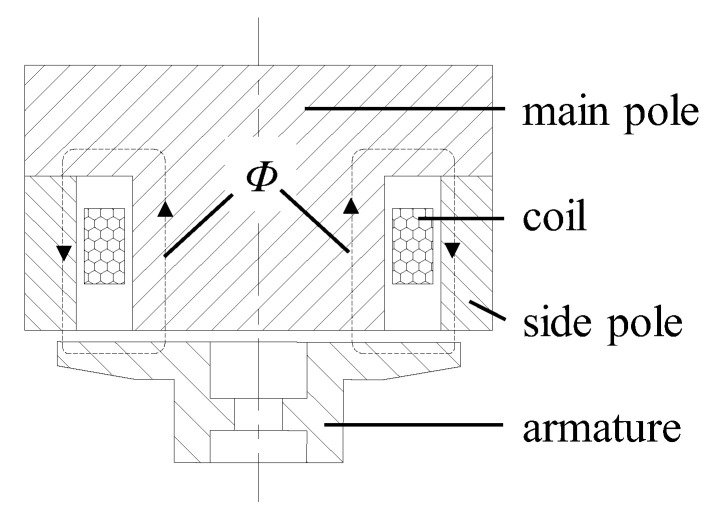
Structure diagram of composite HSV.

**Figure 3:**
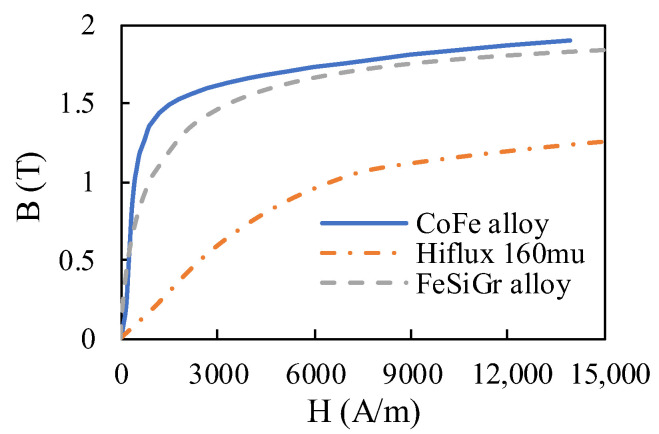
BH curves of soft magnetic materials.

The structure diagram of the conventional HSV is shown in Figure 1. The key components are an iron core, coil, and armature. When the coil is energized, it generates the working magnetic flux *Φ* and closes through the iron core, air gap, and armature. The iron core and armature are magnetized into opposite magnetic poles to produce the electromagnetic force. When the electromagnetic force is greater than the preload force of the reset spring, the armature begins to move toward the iron core. When the coil is powered off, the magnetic field between the iron core and the armature fades, and the armature resets under the action of the reset spring. The structure diagram of the composite HSV is shown in Figure 2. Therein, the iron core is divided into two parts: the main pole and the side pole. In this way, the two soft magnetic materials with different characteristics can be simultaneously used for the iron core. In this paper, the main pole and the side pole are made of Hiflux 160 mu soft magnetic material with high resistivity and low saturation magnetic flux density, and CoFe alloy soft magnetic material with low resistivity and high saturation magnetic flux density, respectively. This strategy can effectively reduce the Eddy current loss of the HSV and consider both the size and growth rate of the electromagnetic force, thereby improving the dynamic response speed of the HSV. Table 1 and Figure 3 list the soft material properties for each component of the conventional and composite HSVs.

## 3. Simulation Model

### 3.1. Model Establishment

**Figure 4:**
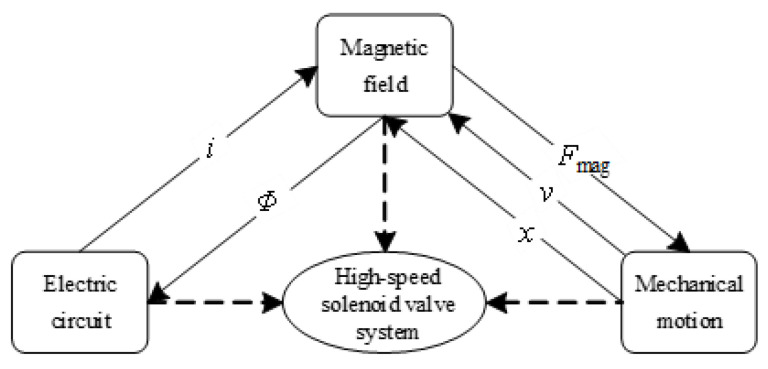
Multi-field coupling relation of HSV.

The dynamic response characteristic of the HSV depends on the comprehensive action of circuit, magnetic field, and mechanical movement. The coupling relationship of each field for the HSV is shown in Figure 4. The circuit provides an excitation current to the magnetic field, which generates an electromagnetic force for the moving part. The displacement and speed of the moving part in turn cause the change in the flux linkage rate, which then causes the change in the excitation current, and so on. The governing equations of each field are as follows:

#### 3.1.1. Governing Equations of Circuit

**Figure 5:**
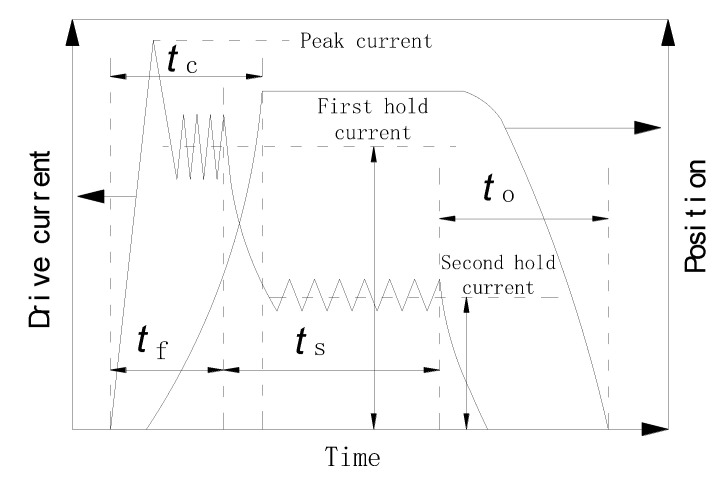
Drive current signal of HSV.

In order to reduce the power consumption of the HSV, the peak-hold drive mode is implemented, as shown in Figure 5. When a drive voltage is applied to the coil, the equivalent circuit equation is as follows:

(1) $$U=Ri+\frac{d\psi }{dt}$$

where *U* denotes the driving voltage [V]; *i* denotes the coil current [A]; *R* denotes the coil resistance [Ω]; *Ψ* denotes the coil flux [Wb]; and *t* denotes the time [s].

#### 3.1.2. Governing Equations of the Magnetic Field

As can be seen from Figure 4, the magnetic field interacts with the circuit and the mechanical motion, and its governing equations are Maxwell’s equations.

(2) $$\left\{\begin{array}{l} \nabla \times H=J+\frac{\partial D}{\partial t}\\ \nabla \times E=-\frac{\partial B}{\partial t}\\ \nabla \cdot D=\rho \\ \nabla \cdot B=0 \end{array}\right.$$

where ***H*** denotes the magnetic field intensity [A/m]; ***J*** denotes the conduction current density [A/m^2^]; ***D*** denotes the electric displacement [C/m]; ***E*** denotes the electric field [V/m]; ***B*** denotes the magnetic flux density [T]; and *ρ* denotes the charge density [C/m^3^].

The expressions of flux linkage and electromagnetic force by the principle of virtual work are shown in Equations (3) and (4), respectively.

(3) Ψ=∫B⋅ds

(4) $$F_{\mathrm{mag}}=\frac{\partial }{\partial z}\left[\int_{V}\left(\int_{0}^{H}B\cdot dH\right)dV\right]$$

where *s* denotes the magnetic flux area [m^2^]; *F*_mag_ denotes the electromagnetic force acting on the displacement direction of the armature [N]; *z* denotes the virtual displacement of the armature [m]; and *V* denotes the space wrapping the armature [m^3^].

#### 3.1.3. Governing Equations of Mechanical Motion

The mechanical motion equation of the HSV is expressed as follows:

(5) $$m\frac{dv}{dt}=F_{\mathrm{mag}}-\lambda v-kx-F_{0}$$

where *m* denotes the mass of the moving part [kg]; *x* denotes the armature displacement [m]; *λ* denotes the damping coefficient [Ns/m]; *k* denotes the stiffness of the return spring [N/m]; and *F*_0_ denotes the preload force of the return spring [N].

The governing Equations (1)~(5) are solved by the time-step finite element method to obtain the dynamic response characteristics of the HSV. In this paper, ANSYS Maxwell and ANSYS Simplorer were employed to conduct the co-simulation for solving the above governing equations [38,39].

### 3.2. Model Verification

**Figure 6:**
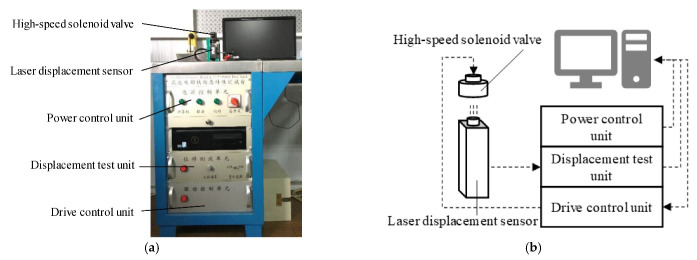
Dynamic characteristic test device of HSV: (**a**) photo image of test device; (**b**) schematic diagram of test device.

**Figure 7:**
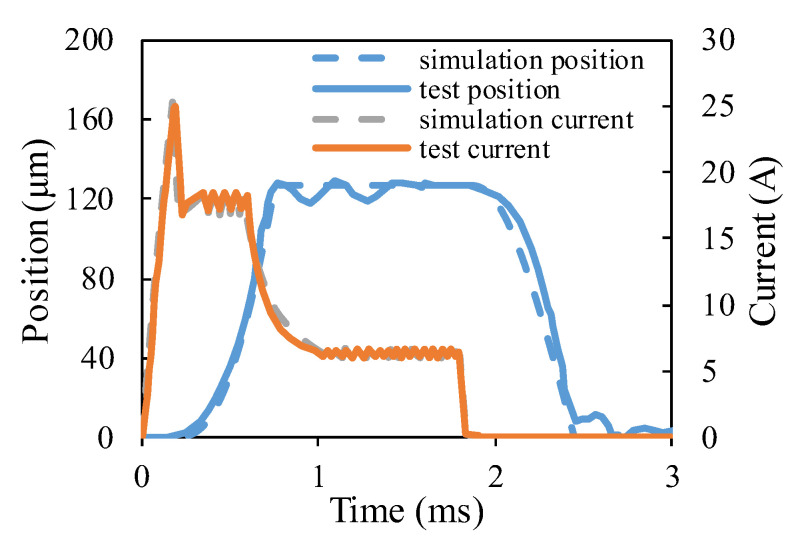
Comparison between the simulation and test results.

The dynamic characteristic test device of the HSV is shown in Figure 6, which was composed of a laser displacement sensor, drive control unit, displacement test unit, industrial computer, power control unit, etc. First, the direction of laser generated by the laser displacement sensor was adjusted to be consistent with the direction of armature movement. Then, the drive control unit provided the corresponding current signal (set by the upper computer software) to the HSV. Under the action of the excitation current, the armature of the HSV moved. So, the laser displacement sensor output the armature displacement signal to the displacement test unit and sent it to the upper computer for display, so that the dynamic response characteristics of the HSV could be obtained. The power control unit provided the electricity for the test system. To ensure the accuracy of simulation results, the actual parameters were used as the simulation boundary conditions. The simulation parameters are shown in Table 2. Figure 7 shows the comparison between the simulation and test results of the conventional HSV. The simulated current curve and displacement curve were in good agreement with the test results, indicating that the simulation model had good prediction accuracy.

## 4. Results and Discussion

### 4.1. Eddy Effect Analysis

**Figure 8:**
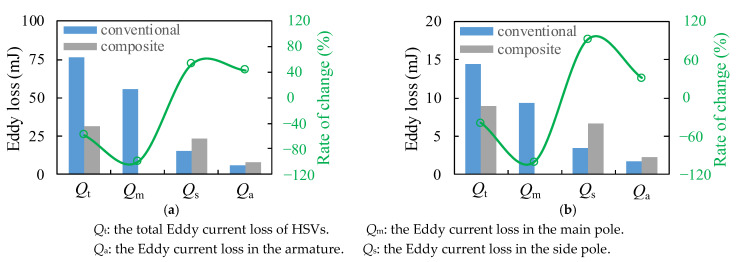
Eddy losses of HSVs: (**a**) in the actuation process; (**b**) in the release process.

**Figure 9:**
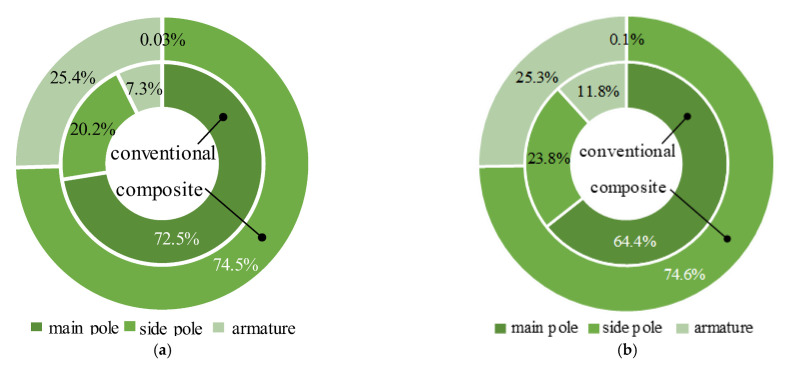
Eddy loss ratio of each component for HSVs: (**a**) in the actuation process; (**b**) in the release process.

As can be seen from Figure 8 and Figure 9, the Eddy current loss of the conventional HSV was mainly concentrated in the main pole; its value in the main pole was 72.5% and 64.4% in the actuation and release processes, respectively. As can be seen from Equation (6) [40], because the main pole of the composite HSV was made of the soft magnetic material with high resistivity, its Eddy effect was significantly weakened. The Eddy current loss of the composite HSV was mainly concentrated in the side pole. The Eddy current loss here was 74.5% and 74.6% during the actuation and release processes, respectively. Compared with the conventional HSV, the total Eddy current loss in the actuation and release processes was reduced by 58.8% and 38.7%, respectively.

(6) $$P_{e}\propto \frac{1}{\sigma }f^{2}B_{m}^{2}$$

where *P*_e_ represents the Eddy current loss power [W]; *σ* denotes the resistivity [Ωm]; *f* denotes the frequency of magnetic field changes [Hz]; and *B*_m_ is the amplitude of the magnetic flux density [T].

**Figure 10:**
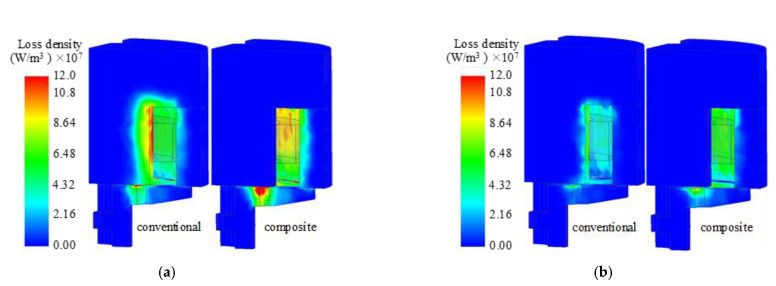
The average Eddy current loss density of HSVs: (**a**) in the actuation process; (**b**) in the release process.

In addition, due to the weakening of the Eddy effect in the main pole of the composite HSV, the magnetic flux density of the side pole and the armature increased at a rate that became higher in the actuation process. The magnetic flux density decreased rapidly in the release process, resulting in an increased Eddy current loss in the two components compared with the conventional HSV. Figure 10 shows the distribution of the average Eddy current loss density of the HSVs in the actuation and release processes.

### 4.2. Dynamic Response Analysis

**Figure 11:**
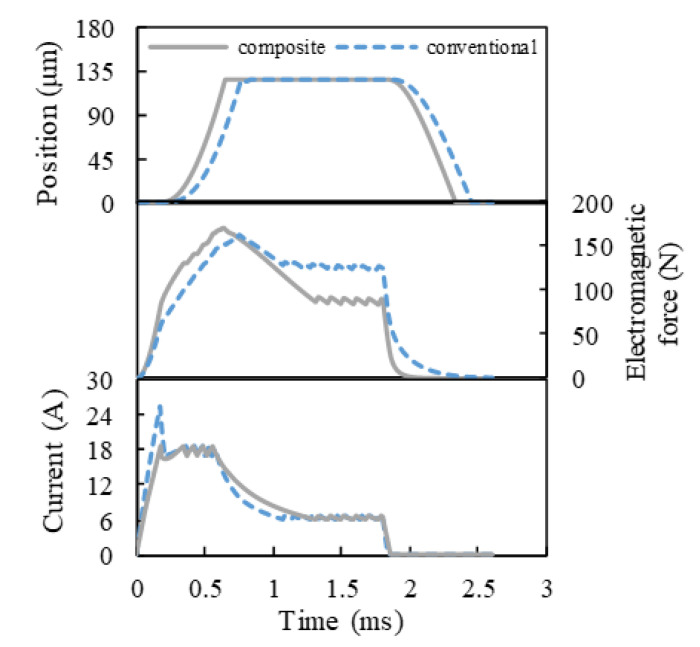
Dynamic response characteristics of HSVs.

**Figure 12:**
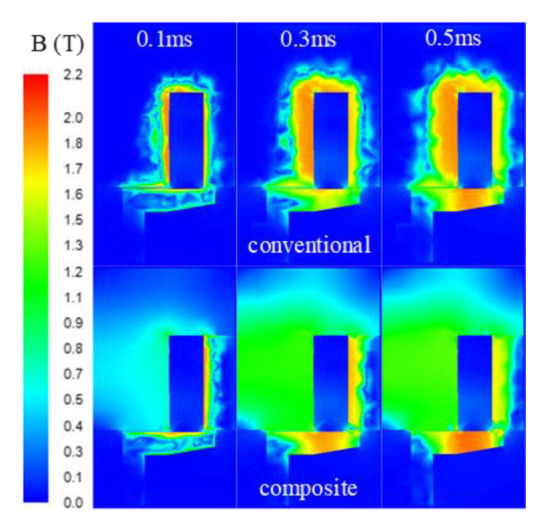
Magnetic flux density on the longitudinal section of HSVs.

As can be seen from Figure 11, compared with the conventional HSV, the dynamic response speed of the composite HSV was significantly improved; its actuation response time *t*_c_ and release response time *t*_o_ were shortened by 15.6% and 18.5%, respectively. The overall Eddy effect of the composite HSV was weakened, which sped up the establishment and decay of the magnetic field (as shown in Figure 12), thus accelerating the increase in and decay rate of the electromagnetic force acting on the armature. Therefore, the dynamic response speed of the composite HSV was improved. On the other hand, because the saturation magnetic flux density of Hiflux 160 mu soft magnetic material is lower than that of CoFe alloy soft magnetic material, it was easier for the magnetic field of the main pole of the composite HSV to approach the saturation state, increasing the overall reluctance of the system. This led to a lower electromagnetic force acting on the armature in the actuation and maintenance stages than that of the conventional HSV. However, it still exceeded the spring preload by 26.9%, which can stably maintain the solenoid valve open.

**Figure 13:**
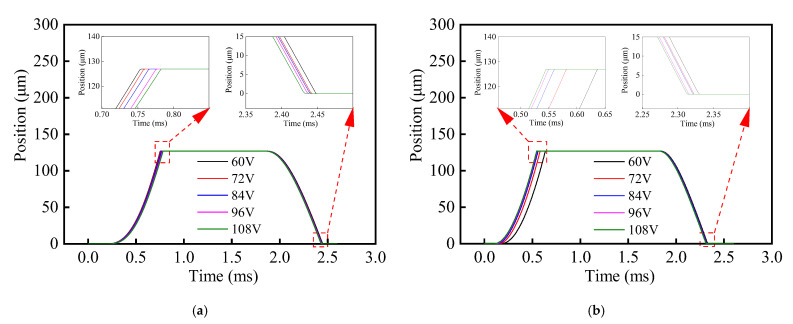
Positions of HSVs at different peak voltages: (**a**) conventional HSV; (**b**) composite HSV.

**Figure 14:**
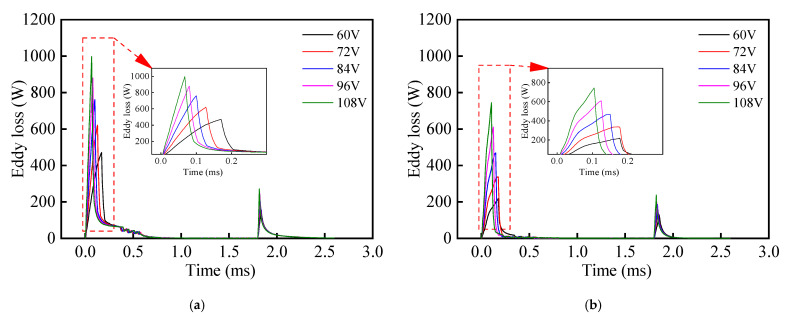
Eddy losses of HSVs at different peak voltages: (**a**) conventional HSV; (**b**) composite HSV.

**Figure 15:**
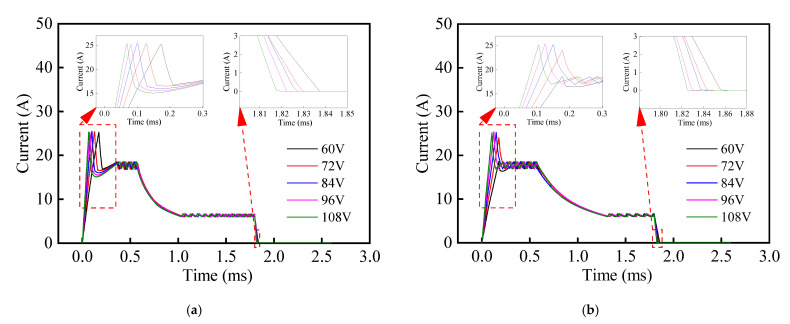
Current signals of HSVs at different peak voltages: (**a**) conventional HSV; (**b**) composite HSV.

**Figure 16:**
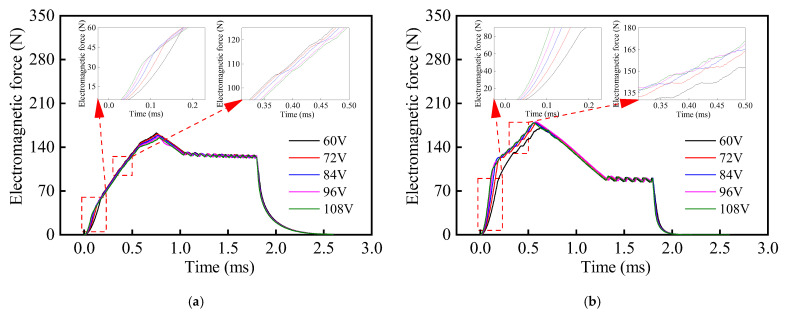
Electromagnetic forces of HSVs at different peak voltages: (**a**) conventional HSV; (**b**) composite HSV.

In addition, it can be seen from Figure 11 that the growth rate of the drive current for the composite HSV was slower than that of the conventional HSV. The rate of current growth can be accelerated by appropriately increasing the drive voltage of the HSV, and thereby, the actuation response speed of composite HSV can be further improved. Figure 13, Figure 14, Figure 15 and Figure 16 show the dynamic response characteristics of the conventional and composite HSVs under different peak voltages. As can be seen from Figure 13, with the increase in peak voltage, the actuation response time of the conventional HSV gradually increased, while the release response time gradually decreased, but the overall impact was not obvious. For the composite HSV, with the increase in peak voltage, the actuation response time began to decrease significantly and then changed not obviously, and the release response time also gradually shortened, but the change was not obvious. This is because, with the increase in the peak voltage, the growth rate of the drive current became faster, so that the growth rate of the electromagnetic force was also faster at the initial stage; at the same time, due to the relatively low resistivity of the soft magnetic material of the iron core for the conventional HSV, a strong Eddy current was generated during the process of rapid current rise, and a higher peak voltage led to a stronger Eddy effect (as shown in Figure 14a). So, the drive current dropped faster when the drive current entered the first hold current stage from the peak current, and the drive current was smaller at the beginning of the first hold current stage (as shown in Figure 15a). Therefore, at the actuation stage, the electromagnetic force of the conventional HSV increased first, and then decreased with the increase in peak voltage, but the difference was not obvious (as shown in Figure 16a). Although the electromagnetic force increased in the early actuation stage, its action time was short, so the actuation response time of the conventional HSV still showed an increasing trend with the increase in peak voltage. For the composite HSV, a soft magnetic material with high resistivity was applied to its iron core, and the Eddy effect generated during the process of rapid current rise was weaker than that of the conventional HSV (as shown in Figure 14); then, the drive current was maintained at the set hold current value when the drive current entered the first hold current stage from the peak current (as shown in Figure 15b). Meanwhile, due to the increase in peak voltage, the rising speed of the current was accelerated, and the Eddy current loss of the composite HSV was relatively small, so that the electromagnetic force during the actuation process increased with the increase in peak voltage (as shown in Figure 16b), and the actuation response time was shortened. Finally, when the coil was powered off, the higher peak voltage resulted in a faster current drop as the coil discharged to the peak voltage source. As a result, with the increase in peak voltage, the electromagnetic force decayed faster, and the release response times of the conventional and composite HSVs gradually decreased.

## 5. Conclusions

In this paper, a novel HSV with a composite iron core is proposed, which combines the characteristics of different soft magnetic materials. The test data showed that the Eddy current loss of the conventional HSV was mainly concentrated in the main pole, while the Eddy current loss of the composite HSV was mainly concentrated in the side pole. Compared with the conventional HSV, the total Eddy current loss of the composite HSV in the actuation and release processes was reduced by 58.8% and 38.7%, respectively. Meanwhile, the dynamic response speed of the composite HSV was significantly improved, and the actuation response time and the release response time were shortened by 15.6% and 18.5%, respectively. In addition, increasing the peak voltage further shortened the actuation response time of the composite HSV, but had no significant effect on the response time of the conventional HSV.

The optimization of the composite HSV should be the object of future studies. Additionally, the design concept of the composite iron core could also be extended to improve other types of solenoid valves.

## Figures and Tables

**Table 1:** Soft material properties of high-speed solenoid valve (HSV).

Soft Magnetic Materials	Properties		Conventional HSV	Composite HSV
	Saturation Magnetic Flux Density *B*_s_ (T)	Electrical Resistivity *σ* (μΩm)		
CoFe alloy	1.9	0.7	iron core	side pole
Hiflux 160 mu [37]	1.5	10,000	-	main pole
FeSiCr alloy	1.8	0.6	armature	armature

**Table 2:** Simulation parameters.

Parameter	Value	Parameter	Value
Peak voltage *V*_P_ (V)	60	Coil turns *N*	40
Hold voltage *V*_h_ (V)	24	Coil resistance *R* (Ω)	0.224
First hold pulse *t*_f_ (ms)	0.58	Mass of the moving part *m* (kg)	0.0245
Second hold pulse *t*_s_ (ms)	1.22	Maximum displacement of armature *x*_max_ (μm)	127
Peak current *I*_p_ (A)	25	Damping coefficient *λ* (Ns/m)	166
First hold current *I*_f_ (A)	18	Stiffness of the return spring *k* (N/m)	33,000
Second hold current *I*_s_ (A)	6	Preload force of the return spring *F*_0_ (N)	62

## Data Availability

Important data are contained within the article. Additional data may be available upon reasonable request to the corresponding author.

## References

[1] Angadi S.V., Jackson R.L. (2022). A critical review on the solenoid valve reliability, performance and remaining useful life including its industrial applications. Eng. Fail. Anal..

[2] Utah M.N., Jung J.C. (2020). Fault state detection and remaining useful life prediction in ac powered solenoid operated valves based on traditional machine learning and deep neural networks. Nucl. Eng. Technol..

[3] Zhong Q., Wang X., Zhou H., Xie G., Hong H., Li Y., Chen B., Yang H. (2022). Investigation into the adjustable dynamic characteristic of the high-speed on/off valve with an advanced pulse width modulation control algorithm. IEEE/ASME Trans. Mechatron..

[4] Jo S., Seo B., Oh H., Youn B.D., Lee D. (2020). Model-based fault detection method for coil burnout in solenoid valves subjected to dynamic thermal loading. IEEE Access.

[5] Gao Q., Wang J., Zhu Y., Wang J., Wang J. (2023). Research status and prospects of control strategies for high speed on/off valves. Processes.

[6] Hung N.B., Lim O. Influences of key design parameters to operating performance of a compressed natural gas injection system using solenoid pressure regulator. Int. J. Engine Res.

[7] Qureshi M.S., Kara B., Ertunc O., Bebek O. Mass flow rate control of solenoid-based injectors. Int. J. Engine Res.

[8] Bai Y., Lan Q., Fan L., Yao J., Kong X., Yang L., Wen L. (2023). Pressure characteristics of the fuel system for two-stroke diesel engines under different operational modes. Fuel.

[9] Bai Y., Lan Q., Fan L., Ma X., Liu H. (2019). Investigation on the fuel injection stability of high pressure common rail system for diesel engines. Int. J. Engine Res..

[10] Liang Y., Liu F., Li Y., An X. (2020). Research on the dynamic cavitation flow characteristics in the control valve region during the opening process of the valve in an electronic unit pump. IEEE Access.

[11] Sun Z., Li G., Wang L., Wang W., Gao Q., Wang J. (2016). Effects of structure parameters on the static electromagnetic characteristics of solenoid valve for an electronic unit pump. Energy Conv. Manag..

[12] Ko S., Song S. (2015). Effects of design parameters on cavitation in a solenoid valve for an electric vehicle braking system and design optimization. J. Mech. Sci. Technol..

[13] Zhao X., Li L., Song J., Li C., Gao X. (2016). Linear control of switching valve in vehicle hydraulic control unit based on sensorless solenoid position estimation. IEEE Trans. Ind. Electron..

[14] Zhao J., Wu X., Song Z., Sun L., Wang X. (2023). Practical hybrid model predictive control for electric pneumatic braking system with on-off solenoid valves. Proc. Inst. Mech. Eng. Part D-J. Automob. Eng..

[15] Safaei M.M., Abedinzadeh R., Khandan A., Barbaz-Isfahani R., Toghraie D. (2023). Synergistic effect of graphene nanosheets and copper oxide nanoparticles on mechanical and thermal properties of composites: Experimental and simulation investigations. Mater. Sci. Eng. B.

[16] Qian W., Vahid M.H., Sun Y., Heidari A., Barbaz-Isfahani R., Saber-Samandari S., Khandan A., Toghraie D. (2021). Investigation on the effect of functionalization of single-walled carbon nanotubes on the mechanical properties of epoxy glass composites: Experimental and molecular dynamics simulation. J. Mater. Res. Technol..

[17] Fan Y., Wang H., Xie L., Hu N., Yang J. (2023). Armature structure optimization of annular multipole solenoid valves based on electromagnetic force distribution. Actuators.

[18] Zhang B., Zhong Q., Ma J., Hong H., Bao H., Shi Y., Yang H. (2018). Self-correcting pwm control for dynamic performance preservation in high speed on/off valve. Mechatronics.

[19] Hung N.B., Lim O. (2019). Improvement of electromagnetic force and dynamic response of a solenoid injector based on the effects of key parameters. Int. J. Automot. Technol..

[20] Yang L., Gao T., Du X., Zhai F., Lu C., Kong X. (2022). Electromagnetic characteristics analysis and structure optimization of high-speed fuel solenoid valves. Machines.

[21] Li T., Zhang Y., Liang Y., Yang Y., Jiao J. (2021). Multiobjective optimization research on the response time of a pneumatic pilot-operated high speed on/off valve. Int. J. Appl. Electromagn. Mech..

[22] Zhao J., Shi Y., Grekhov L., Ma X. (2017). Effects of structure parameters on the static electromagnetic characteristics of high speed solenoid valves. Int. J. Appl. Electromagn. Mech..

[23] Wang L., Li G., Xu C., Xi X., Wu X., Sun S. (2016). Effect of characteristic parameters on the magnetic properties of solenoid valve for high-pressure common rail diesel engine. Energy Conv. Manag..

[24] Liu P., Fan L., Zhou W., Ma X., Song E. (2017). Dynamic performances analysis and optimization of novel high-speed electromagnetic actuator for electronic fuel injection system of diesel engine. J. Mech. Sci. Technol..

[25] Zhao J., Zirka S.E., Moroz Y.I. (2021). Duality-derived models of high-speed electromagnetic valves. IEEE Trans. Ind. Electron..

[26] Zhao J., Yue P., Wei K. (2020). Eddy current effects on the dynamic response of high-speed solenoid valve for common rail injector. Int. J. Appl. Electromagn. Mech..

[27] Bai Y., Chen X., Liu P., Fan L., Pu D., Ma X. (2022). Research on eddy-current loss of a permanent magnet high-speed solenoid valve. Trans. CSICE.

[28] Tan C., Li B., Liu Y., Ge W., Sun B. (2019). Multiphysics methodology for thermal modelling and quantitative analysis of electromagnetic linear actuator. Smart Mater. Struct..

[29] Zhao J., Wang M., Wang Z., Grekhov L., Qiu T., Ma X. (2017). Different boost voltage effects on the dynamic response and energy losses of high-speed solenoid valves. Appl. Therm. Eng..

[30] Zhao J., Yue P., Grekhov L., Ma X. (2018). Hold current effects on the power losses of high-speed solenoid valve for common-rail injector. Appl. Therm. Eng..

[31] Cheng Q., Zhang Z., Xie N. (2016). Power losses analysis of the gasoline direct injector within different driven strategies. Int. J. Appl. Electromagn. Mech..

[32] Cheng Q., Zhang Z., Xie N. (2015). Power losses and dynamic response analysis of ultra-high speed solenoid injector within different driven strategies. Appl. Therm. Eng..

[33] Zhong Q., Wang X., Xie G., Yang H., Yu C., Xu E., Li Y. (2021). Analysis of dynamic characteristics and power losses of high speed on/off valve with pre-existing control algorithm. Energies.

[34] Cheng Q., Zhang Z., Guo H., Xie N. (2014). Improved processing and performance of GDI injector based on metal injection molding technology. Int. J. Appl. Electromagn. Mech..

[35] Dai J., Zhao Z., Xu S., Wang C., Zhu J., Fan X. (2019). Inhibition of iron loss of the inner yoke in electromagnetic linear actuator. IET Electr. Power Appl..

[36] Fohr F., Volbers N., Gerster J. Soft magnetic materials tailored for efficient fuel injection. Proceedings of the Magnetic Materials for the 21st Century.

[37] Magnetics—Powder Cores Manufacturer. https://www.mag-inc.com/Products/Powder-Cores/High-Flux-Cores?lang=en-US.

[38] (2020). ANSYS Electromagnetics Suite 2020R1, Maxwell Help.

[39] (2020). ANSYS Electromagnetics Suite 2020R1, Twin Builder Help.

[40] Cheng Q., Zhang Z., Guo H., Xie N. (2014). Simulation and analysis on electro-magnetic-thermal coupling of solenoid GDI injector. Int. J. Appl. Electromagn. Mech..

